# Intravital imaging tumor screen used to identify novel metastasis-blocking therapeutic targets

**DOI:** 10.15698/cst2018.10.159

**Published:** 2018-10-04

**Authors:** Konstantin Stoletov, Lian Willetts, Perrin H. Beatty, John D. Lewis

**Affiliations:** 1 Department of Oncology, University of Alberta, Edmonton, Alberta, Canada, T6G 2E1.

**Keywords:** ex ovo chick embryo model, fluorescence time-lapse intravital imaging, high throughput sequencing, intravasation, metastaticc cancer, shRNA library, therapeutic targets

## Abstract

Cancer cell motility is a key driver of metastasis. Although the intravasation of cancer cells into the blood stream is highly dependent on their motility and metastatic dissemination is the primary cause of cancer related deaths, current therapeutic strategies do not target the genes and proteins that are essential for cell motility. A primary reason for this is because the identification of cell motility-related genes that are relevant *in vivo* requires the visualization of metastatic lesions forming in an appropriate *in vivo *model. The cancer research community has lacked an *in vivo* and intravital metastatic cancer model that could be imaged as motility developed, in real-time. To address this, we developed a novel quantitative *in vivo* screening platform based on intravital imaging in shell-less *ex ovo* chick embryos. We applied this imaging approach to screen a human genome-wide short hairpin RNA library (shRNA) versus the highly motile head and neck cancer cells (HEp3 cell line) introduced into the chorioallantoic membrane (CAM) of chick embryos and identified multiple novel *in vivo* cancer cell motility-associated genes. When the expression of several of the identified genes was inhibited in the HEp3 tumors, we observed a nearly total block of spontaneous cancer metastasis.

## INTRODUCTION

Currently, many cancer patients undergo chemotherapy, which is aimed at mitigating the risk of future metastasis. These treatments, however, have numerous system-wide off-target effects that negatively impact the patients’ quality of life. Although cancer cells are not recognized by the host immune system as "other" and so cannot be depended on to potentiate an immunogenic response, cancer cells do differentially express genes compared to normal cells. We’ve found that this is the case *in vivo* with cancer cell motility-related genes. Given that the genes and signaling networks that drive *in vivo* motility and intravasation are different from those required for efficient primary tumor formation we sought to develop an *in vivo* approach to feasibly screen for genes required for motility, and therefore intravasation and metastasis. Metastasis is dependent on a tumor cell’s ability to intravasate, disseminate, evade immune detection, survive, proliferate, colonize elsewhere in the host, and do this repeatedly. We were able to visualize tumor colonies with expression-inhibited cell motility genes, and found that they form compact colonies instead of diffuse ones. This *in vivo* colony phenotype was distinctive enough to utilize this approach to screen for therapeutic targets of cell motility that would in turn impact intravasation and metastasis. Using our three-dimensional, intravital tumor-development screening approach in chick embryos to identify functional metastatic genes showed the importance of using an *in vivo* microenvironment because these phenotypes are not well recapitulated in two-dimensional culture.

## INTRAVITAL IMAGING WITH *EX OVO* CHICK EMBRYOS AND IDENTIFYING METASTASIS GENES BY SCREENING A GENOME-WIDE RNA LIBRARY IN HEp3 CELLS

Structurally, the chicken embryo CAM is a highly vascularized, thin, transparent organ, surrounded by a dense collagen-rich matrix, very similar to mouse lung tissue. Human cancer cells robustly interact with the collagen fiber network in both tissues. These features make the CAM very advantageous for human cancer cell tumor development because it can be easily visualized using *in vivo* multiphoton imaging.

**Figure 1:**
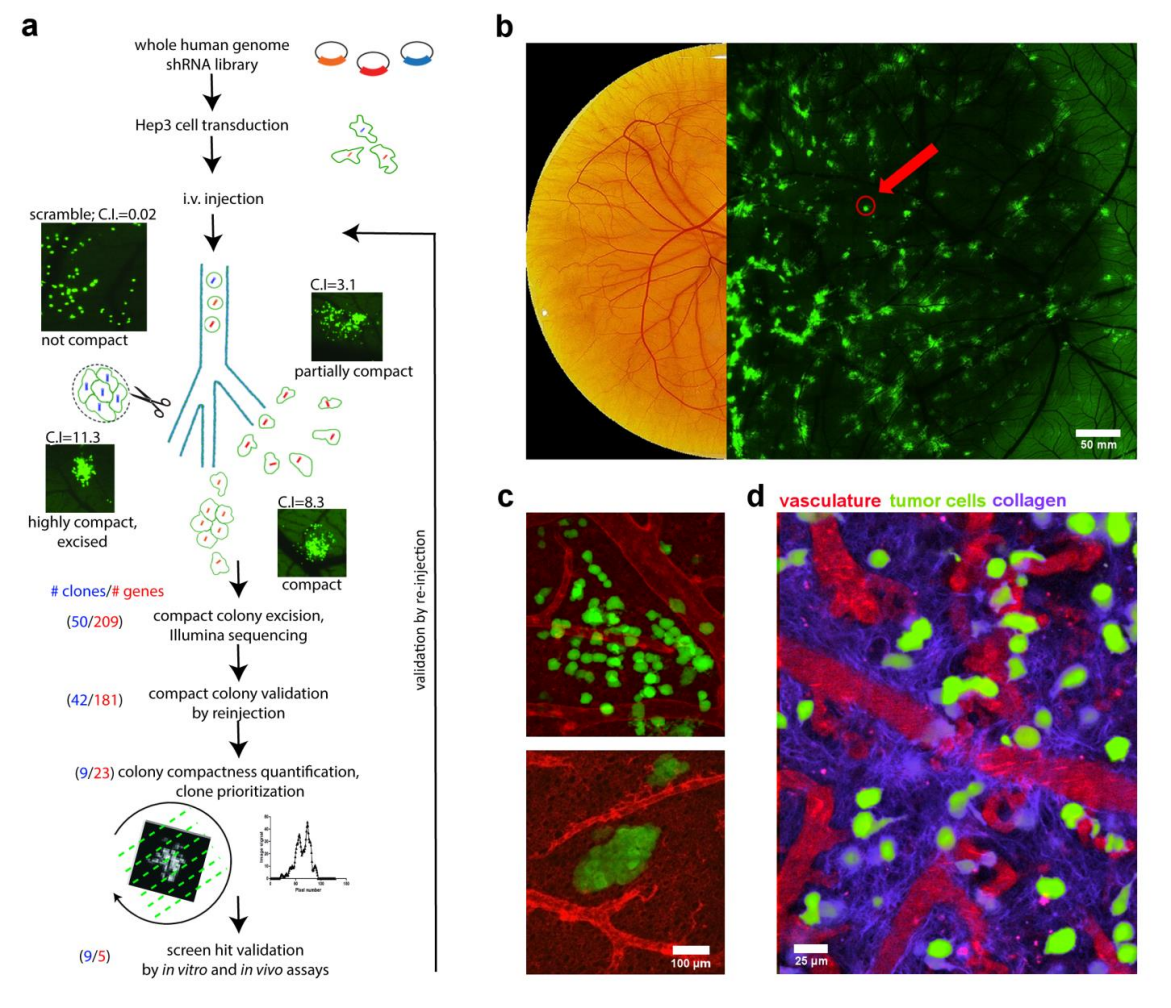
FIGURE 1: Chicken embryo as a platform for anti-metastatic target discovery. **(a)** Overview of intravital shRNA screen for drivers of human cancer metastasis. **(b)** Chicken CAM, fifteen days post fertilization. Left image shows brightfield view, right image shows a low-magnification, fluorescence image of GFP-expressing metastatic cancer cell colonies within the CAM tissue. Red arrow points to compact metastatic cancer cell colony. **(c)** High-resolution images of invasive (upper) and compact (lower) metastatic cancer cell colonies (green) and their associated vasculature (red) within the chicken CAM tissue. **(d)** Intravital imaging of cancer cells (green) and their interaction with vasculature (red) and the collagen matrix (purple) within the chicken CAM tissue. Collagen was visualized using second harmonic generation (SHG) imaging.

In our work, HEp3 cells were transformed with the 79 805-shRNA vector library so that each HEp3 cell contained a single shRNA vector. The transformed HEp3 cancer cells were then intravenously injected into the chick embryo where they disseminated throughout the vasculature. Roughly 10% of the 25,000 injected tumor cells arrested individually in the CAM and extravasated into the extravascular stroma. They then proliferated into isolated, invasive metastatic colonies, each arising from a single transformed cell (**Figure 1a**).

Because thousands of individual metastatic colonies are visualized simultaneously in the CAM of a single embryo, we found that it was feasible to screen large libraries of genes by identifying phenotypic changes using intravital microscopy. To screen the whole genome shRNA library containing 79,805 sequence-verified shRNAs targeting 30,728 human genes, we estimated that conducting the screen in 100 embryos would give us a minimum 3x coverage of the entire library.

## METASTATIC AND BLOCKED METASTATIC COLONY PHENOTYPES

HEp3 cells are highly motile and as they proliferate in the CAM they form a "spread out" morphology because the cells migrate a significant distance from the point of extravasation. When their motility is reduced, however, the resulting colonies adopt a more compact morphology that was easily distinguished from those formed from highly motile HEp3 tumor cells. Since the *ex ovo* chick embryo CAM is readily accessible, these compact tumour cell colonies were readily excised from the surrounding tissue for further analysis (**Figure 1b, c**). We utilized a high throughput sequencing approach to characterize the shRNAs present in each compact colony and identified eleven unique shRNA-targeted genes (ACTB, ACTC1, APBA2, C14orf142, KB-1460A1.5, KDELR3, KIAA0922, KIF3B, NR2F1, SRPK1, and TMEM229B) whose expression is associated with *in vivo* cancer cell motility.

## CANCER CELL-COLLAGEN MATRIX INTERACTION

When cancer cells invade into the stroma surrounding a tumor, they must first actively reorganize the collagen rich matrix at the tumor front to create areas of densely bundled, aligned collagen fibers that can be exploited as motility pathways. We surmised that our screen-identified genes, identified by their requirement for cancer cell motility, might be involved in the coordination of directional *in vivo* cell migration and invasion through collagen remodelling. Time-lapse imaging of the primary tumor fronts showed that control HEp3 cells actively invade along aligned collagen bundles perpendicular to the tumor primary front (**Figure 1d**). Inhibition of screen-identified genes such as KIF3B resulted in an inability to reorganize the collagen fiber network at the primary tumor periphery in the chick embryo CAM, resulting in an almost complete absence of collagen bundles. Accordingly, invasion of the KIF3B-mutant cell lines was significantly reduced when visualized in a 3D collagen invasion assay. From this, we hypothesized that genes required for *in vivo* cell motility and directional cell migration would also be required for spontaneous metastasis.

## IDENTIFICATION OF NOVEL *IN VIVO* CANCER CELL MOTILITY AND METASTASIS GENES

We then explored the physiological relevance of the screen-identified genes in human cancer progression and metastasis. The top hit genes identified in our screen were found to be significantly upregulated in the metastatic lesions of several solid cancer types including: melanoma, prostate cancer, head and neck cancer, lung cancer, ovarian cancer and colon cancer. In addition, several of the genes identified in this screen were linked previously with cancer cell invasion and/or migration, which validated our *in vivo*, intravital imaging approach.

We found that several of the top eleven gene hits had previously-described functions, but had not been shown previously to be associated with metastasis. Others have unknown function. Identification of KIF3B as a top metastasis target is intriguing since this gene is part of the kinesin motor machinery responsible for transporting multiple therapeutically relevant molecules such as β-catenin and MT1-MMP to the cancer cell front. KIF3B has been shown to be essential for collagen fiber matrix remodeling, which is necessary for intravasion and ultimately metastasis. This is consistent with our finding that inhibiting KIF3B expression efficiently inhibits these processes and blocks cancer cell metastasis. The identification of the actin isoforms ACTB and ACTC1 in our screen was not surprising because they are known to be central in cell migration machinery and have been previously identified using *in vitro* screening approaches. Another hit, SRPK1, is a VEGF splice regulator previously linked to cancer progression and metastasis. While we observed that reduction of SRPK1 expression was linked with a compact colony phenotype, we did not observe an inhibition of primary tumor growth. SRPK1 was also recently identified as a cell migration-mediator using an *in vitro* screening approach, which, together with our results, supports the idea that SRPK1 plays a more direct mechanistic role in cell migration and metastasis. These various findings highlight the importance of identifying the overlapping mechanisms responsible for productive cell migration *in vivo* compared to *in vitro*.

We also identified several genes and non-coding RNAs of unknown function in this screen, suggesting a yet-to-be-described role in cancer progression and metastasis. Certainly, the identification of pro-metastatic genes and RNAs warrant future investigation and potential development as potential therapeutic targets.

## CONCLUSIONS

Overall, we found an extraordinarily tight link between a gene’s *in vivo* cancer cell motility phenotype and its requirement for successful metastatic spread. This demonstrates that our quantitative *in vivo* imaging-based screening approach is a powerful tool to identify potential therapeutic targets for metastasis, paving the way for the development of new therapies to block this deadliest aspect of human cancer. By coupling the highly accessible *ex ovo* chick embryo tumor model with fluorescence time-lapse intravital imaging and high-throughput sequencing, we designed a rapid and quantitative method to detect clinically-relevant metastasis phenotypes. In particular, three of the genes discovered using this intravital imaging platform, KIF3B, SRPK1 and NR2F1, represented very promising therapeutic targets for metastasis. Future work would be to test these metastasis-associated genes and gene-products as drug targets aimed at blocking metastasis *in vivo*.

Therapies designed to specifically target the rate limiting steps of metastatic dissemination of tumor cells could significantly improve cancer treatment by directly blocking the systemic spread of cancer. Cancer treatment could also shift away from the use of chemotherapies with detrimental side-effects which would dramatically improve the quality of life of cancer patients.

